# Multivariate analysis of associations between clinical sequencing and outcome in glioblastoma

**DOI:** 10.1093/noajnl/vdac002

**Published:** 2022-01-10

**Authors:** Peter H Yang, Yu Tao, Jingqin Luo, Mounica Paturu, Hsiang-Chih Lu, Shakti Ramkissoon, Jonathan W Heusel, Eric C Leuthardt, Michael R Chicoine, Joshua L Dowling, Gavin P Dunn, Eric Duncavage, Sonika Dahiya, Arindam R Chattherjee, Albert H Kim

**Affiliations:** 1 Department of Neurological Surgery, Washington University School of Medicine in St. Louis, St. Louis, Missouri, USA; 2 Department of Pathology and Immunology, Washington University School of Medicine in St. Louis, St. Louis, Missouri, USA; 3 Department of Surgery, Public Health Sciences Division, Washington University School of Medicine in St. Louis, St. Louis, Missouri, USA; 4 Department of Genetics, Washington University School of Medicine in St. Louis, St. Louis, Missouri, USA; 5 Foundation Medicine Inc., Cambridge, Massachusetts, USA; 6 Brain Tumor Center, Siteman Cancer Center, Washington University School of Medicine in St. Louis, St. Louis, Missouri, USA; 7 Mallinckrodt Institute of Radiology, Washington University School of Medicine in St. Louis, St. Louis, Missouri, USA

**Keywords:** DNA, glioblastoma, high-throughput nucleotide analysis, multivariate analysis, retrospective studies, sequence analysis

## Abstract

**Background:**

Many factors impact survival in patients with glioblastoma, including age, Karnofsky Performance Status, postoperative chemoradiation, *IDH1/2* mutation status, *MGMT* promoter methylation status, and extent of resection. High-throughput next-generation sequencing is a widely available diagnostic tool, but the independent impact of tumors harboring specific mutant genes on survival and the efficacy of extent of resection are not clear.

**Methods:**

We utilized a widely available diagnostic platform (FoundationOne CDx) to perform high-throughput next-generation sequencing on 185 patients with newly diagnosed glioblastoma in our tertiary care center. We performed multivariate analysis to control for clinical parameters with known impact on survival to elucidate the independent prognostic value of prevalent mutant genes and the independent impact of gross total resection.

**Results:**

When controlling for factors with known prognostic significance including *IDH1/2* mutation and after multiple comparisons analysis, *CDKN2B* and *EGFR* mutations were associated with reduced overall survival while *PTEN* mutation was associated with improved overall survival. Gross total resection, compared to other extent of resection, was associated with improved overall survival in patients with tumors harboring mutations in *CDKN2A*, *CDKN2B*, *EGFR*, *PTEN*, *TERT* promoter, and *TP53*. All patients possessed at least one of these 6 mutant genes.

**Conclusions:**

This study verifies the independent prognostic value of several mutant genes in glioblastoma. Six commonly found mutant genes were associated with improved survival when gross total resection was achieved. Thus, even when accounting for known predictors of survival and multiple mutant gene comparisons, extent of resection continues to be strongly associated with survival.

**Key Points**

- Complete resection of tumors with any of 6 common mutant genes improves survival.
- Multivariate and multiple comparisons analyses are critical in sequencing studies.

**Importance of the Study**

This study underscores the importance of utilizing multivariate analysis while taking into account multiple comparisons when determining the clinical significance of a large panel of genetic mutations in glioblastoma. First, our findings verify the prognostic value of several previously reported mutant genes with increased scientific rigor. Second, we find that gross total resection is beneficial for patients with tumors harboring any of 6 common mutant genes. A rigorous analytical approach is important to control for the heterogeneous clinical and genetic profiles of patients with glioblastomas, which is critical for patient selection in translational neuro-oncology research.

Glioblastoma is the most common primary malignant central nervous system tumor in adults. Median survival for patients with newly diagnosed glioblastoma is approximately 20 months, and 5-year survival remains poor at 5%.^1–3^ Prior to the era of clinical genetic sequencing, several factors had been known to be associated with prolonged overall survival (OS) including higher initial Karnofsky Performance Status (KPS), younger age, extent of standard-of-care adjuvant chemoradiation, and higher extent of tumor resection (EOR).^4–6^ Knowledge of these factors has paved the way for establishing the standard of care for newly diagnosed glioblastoma—maximal safe resection followed by radiotherapy and concurrent temozolomide.

Modern clinical genetic testing in glioblastoma has led to further improvements in the classification of glioblastoma into several molecular subgroups, which were found to have prognostic and potentially therapeutic significance.^7^ Two molecular features with particularly significant clinical implications are the isocitrate dehydrogenase 1 and 2 (*IDH1* and *IDH2*) gene mutations and *O*^6^-methylguanine-DNA-methyltransferase (*MGMT*) promoter methylation. *IDH1/2* mutations are found in nearly 80% of secondary glioblastoma (also referred to as IDH-mutant glioblastoma) and in approximately 5% of de novo glioblastoma and are associated with better prognosis.^8,9^ The DNA repair gene *MGMT* can be epigenetically silenced through promoter methylation, which renders tumors more susceptible to alkylating agents such as temozolomide. *MGMT* promoter methylation is more common in *IDH1/2*-mutant glioblastoma and is associated with a better prognosis in patients receiving temozolomide.^10^ Glioblastoma has been shown to have a highly heterogeneous genetic profile beyond the *IDH1/2* mutation and *MGMT* promoter methylation, but with few exceptions, surprisingly little is known about the prognosis of patients with tumors harboring other genetic mutations. Even among the few reported prognostic mutant genes, the literature contains conflicting findings.^11–13^ The prognostic significance of *EGFRvIII* is controversial^14,15^; for example, *PTEN* mutation has been associated with poor survival in glioma patients.^16^*CDKN2A* and *CDKN2B* deletions, but not *TERT* mutation or *EGFR* amplification, have been recently reported to be an independent prognostic marker for *IDH1/2*-wildtype glioblastoma.^17^ Few studies investigating the prognostic value of particular gene alterations have taken into account factors that are known to impact survival. One study investigated the prognostic significance of *TERT* promoter mutation.^18^ The authors demonstrated in a univariate analysis that age, KPS, EOR, *MGMT* promoter methylation status, and postoperative chemoradiation were prognostic for survival. In a subsequent multivariate analysis, the authors found that *TERT* promoter mutation was an independent predictor of poor prognosis. In their exploratory analysis, the authors found that *TERT* promoter mutation may be prognostic only if subtotal resection (STR) was achieved and if patients did not undergo adjuvant chemotherapy, leading to the conclusion that patients with *TERT* promoter mutations may require aggressive treatment.

Knowledge of the relationship between particular genetic events in a tumor and EOR have implications for how aggressively tumors should be resected. For example, in a study of 282 patients with newly diagnosed anaplastic astrocytomas or glioblastomas stratified by *IDH1* mutation status, patients with tumors harboring *IDH1* mutations had an additional survival benefit if further resection of the nonenhancing disease (supramaximal resection) in addition to the enhancing disease was achieved.^19^ More recently, a survival benefit was demonstrated in younger patients with even *IDH1*-wildtype glioblastomas after supramaximal resection.^20^ In theory, however, more discrete knowledge of genetic subtypes within the main subgroups of *IDH1*-wildtype and -mutant glioblastomas could influence the impact of EOR, and other practices, and ultimately affect patient survival. Therefore, there is an urgent need to study the independent prognostic significance of EOR on the known genetic subtypes of glioblastoma by controlling for factors with a known impact on survival. Moreover, while it is known that gross total resection (GTR) positively impacts survival, it is not known to what degree this is true when stratifying by individual mutant genes.

With the knowledge of factors with known prognostic significance and the availability of high-throughput next-generation sequencing (NGS), there is an opportunity to study the independent prognostic significance of multiple known mutant genes in glioblastoma. In this retrospective cohort study, we used age, KPS, extent of adjuvant chemoradiation, *MGMT* promoter methylation status, EOR, and *IDH1/2* mutation status as covariates while accounting for multiple gene comparisons to investigate this hypothesis. We also explored the independent impact of GTR on survival in tumors with the most prevalent mutant genes. Ultimately, genetic tumor classification can be utilized as a molecular diagnostic tool or a prognostic indicator of OS, further leading to the development and characterization of new treatment strategies.

## Material and Methods

### Patient Selection

The study was conducted with the approval of the institutional review board. Patients aged 18 and older with newly diagnosed *IDH1/2*-wildtype and -mutant glioblastoma (WHO 2016 criteria), who were treated with surgery at our tertiary center and had samples sent to Foundation Medicine between 2015 and 2020, were retrospectively reviewed. All pathology slides were reviewed for confirmation of diagnosis of glioblastoma by a board-certified neuropathologist (S.D.). Exclusion criteria included patients treated at another facility whose adjuvant chemoradiation regimen was unknown, secondary glioblastoma (prior low-grade or anaplastic astrocytoma), secondary debulking or ablative surgery after recurrence, patients lost to follow-up after surgery, post-resection MRI performed greater than 72 h after surgery, and patients who died within 60 days after surgery to exclude confounding variables affecting survival such as sepsis and acute cardiopulmonary events. To determine *MGMT* promoter methylation status, DNA was isolated from formalin-fixed, paraffin-embedded tumor tissue specimens. Molecular analysis of the *MGMT* gene was performed by methylation-specific PCR and detected on ABI 7900 (Labcorp Center for Molecular Biology and Pathology). The *MGMT* and beta-actin copy numbers were used to calculate the ratio of the *MGMT*/beta-actin ×1000. This assay detects the target gene at 45–50 copies per reaction. Methylation score ≥2.00 was considered positive for promoter methylation.

### Next-Generation Sequencing

NGS was exclusively performed using the FDA-approved, commercially available FoundationOne CDx test (F1CDx Foundation Medicine), which is a Clinical Laboratory Improvement Amendments (CLIA)-certified in vitro diagnostic test that uses targeted high-throughput hybridization-based capture technology to detect substitutions, insertions, deletion, and copy number alterations in 324 genes and select gene rearrangements using DNA isolated from formalin-fixed paraffin-embedded tumor tissue specimens (https://info.foundationmedicine.com/hubfs/FMI%20Labels/FoundationOne_CDx_Label_Technical_Info.pdf). This sequencing platform has been used routinely at our institution since 2015 as an additional tool to further characterize the molecular changes within central nervous system neoplasms.

### Treatment and Outcome

**Figure 1.**
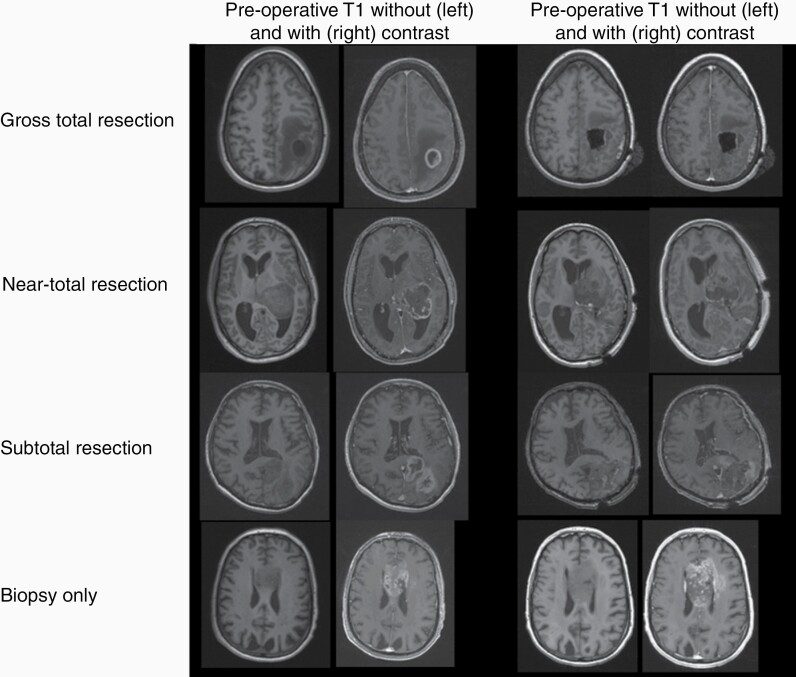
Extent of resection as depicted by T1-weighted sequences without and with gadolinium contrast on preoperative and postoperative magnetic resonance imaging.

Clinical parameters collected included age, EOR, progression-free survival (PFS; defined as the time between the date of surgery and the date of the MRI read as concerning for recurrence), OS (defined as the time between the date of surgery and the date of hospice or death), baseline KPS as determined by first postoperative oncology visit, *MGMT* promoter status, and the extent to which patients received adjuvant chemoradiation (full, partial, or none). All patients underwent standard of practice postoperative 3T brain MRI (including a slice thickness 1.0 mm MPRAGE sequence) without and with gadolinium contrast within 72 h of surgery. EOR for all cases was independently assessed by a subspecialty board-certified neuroradiologist (A.R.C.) with greater than 7 years of experience based on RANO criteria.^21,22^ The assessments were compared to the original neuroradiology report for concordance. Discrepancies between central review and neuroradiology reports were reconciled by direct review of images by another author (P.H.Y.) and resulted in an agreement with a central review. We defined postoperative MRI GTR as 100% complete resection of enhancing tissue, near-total resection (NTR) as resection of between approximately 80–99% of enhancing tissue (less than 5 cm^3^ residual contrast-enhancing tissue volume), and STR as resection of less than 80% (greater than 5 cm^3^ residual contrast-enhancing tissue volume) (Figure 1). Any areas of intrinsic T1 hyperintensity representing postoperative blood products were distinguished and excluded from true enhancing tissue assessment. Biopsy only was also included and was defined as a needle biopsy without tumor debulking via craniotomy.

### Statistics

**Figure 2.**
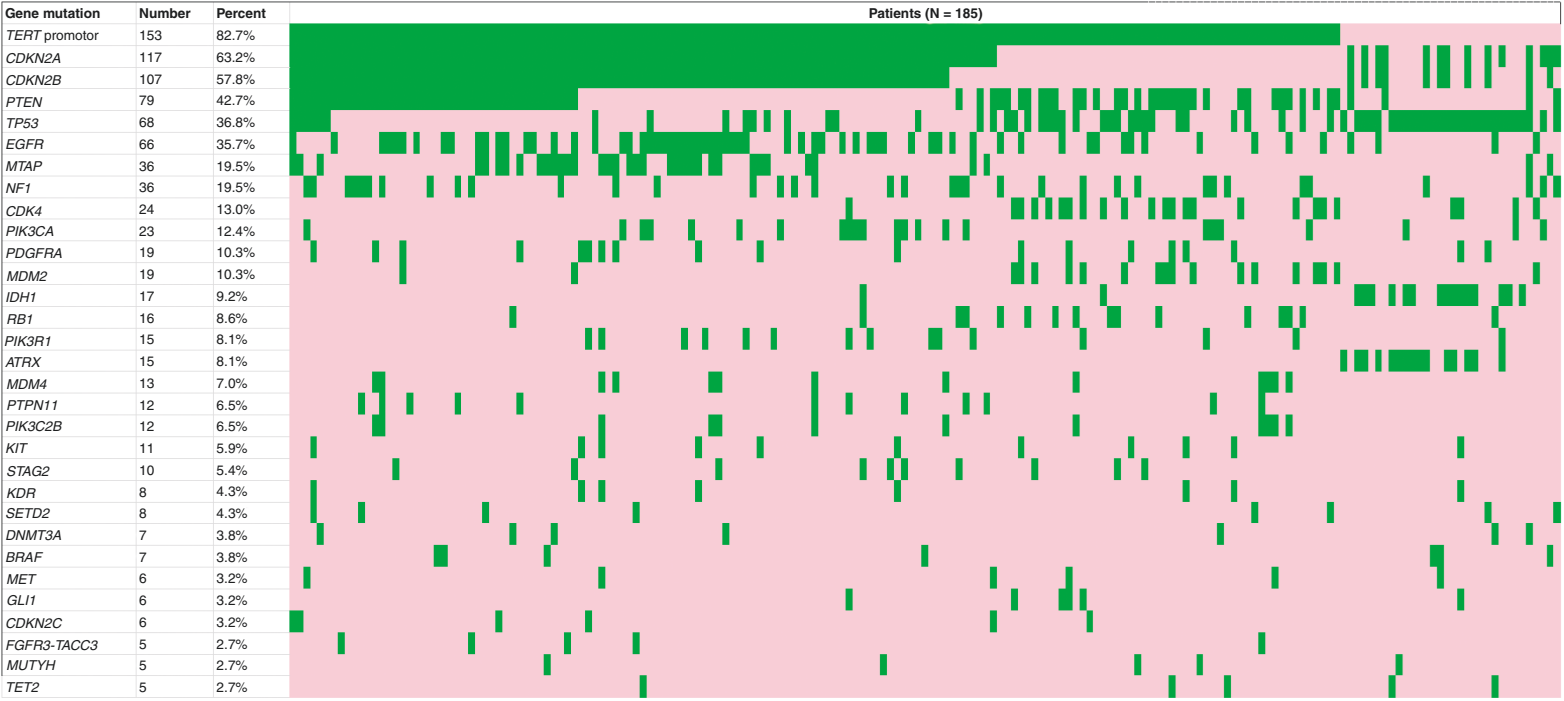
Waterfall plot depicting the most commonly mutated genes in our cohort, sorted by frequency. The most commonly mutated genes included *TERT* promoter (82.7%), *CDKN2A* (63.2%), *CDKN2B* (57.8%), *PTEN* (42.7%), *TP53* (36.8%), and *EGFR* (35.7%). *IDH1* mutation frequency was 9.2%. Mutated genes are depicted in green and non-mutated genes are depicted in red.

Kaplan–Meier (KM) analysis was conducted to generate survival curves for PFS and OS of parameters with previously known impact on survival as a validation of our patient cohort (Figure 2). The log-rank test was used to examine the statistical significance of the differences observed between the groups. A prognostic study was performed using a multivariate Cox proportional hazards model to compute hazard ratios (HRs) and 95% confidence intervals of the most frequently mutated tumor genes while controlling for covariates with known impact on survival. A multivariate analysis was performed to investigate the impact on survival of GTR versus all other EOR in patients with tumors that harbored the most frequently mutated genes. The Benjamini–Hochberg procedure controlling false discovery rate (FDR) at a 5% level was used to adjust the survival analysis. The 2-tailed *t* test with *P* < .05 was considered statistically significant. All analyses were performed within Statistical Analysis System (SAS, version 9.4; SAS Institute Inc.).

### Exploratory Validation Analysis

As an exploratory validation, we collected the clinical and radiological data as above and performed an identical analysis of glioblastoma patients who underwent surgery between 2011 and 2015 at our institution. All specimens underwent previously validated NGS testing through an institutional platform.^23–25^ We used the bootstrapping technique on the FoundationOne data set to perform a power analysis (Supplementary Methods, Supplementary Table 1).

## Results

### Patient Characteristics

About 269 patients were screened and 185 patients (108 male, 58.4%) with a median age of 62.2 years (range 23.3–84.6 years) met criteria for further analysis. About 87.0% of patients (N = 161) had a postoperative KPS of 70 or higher. 9.7% of patients (N = 18) had *IDH1* or *IDH2* mutation and 42.4% (N = 75 out of 177 with completed results) had *MGMT* promoter methylation. 27.0% (N = 50) patients underwent GTR, 10.3% (N = 19) underwent NTR, 35.1% (N = 65) underwent STR, and 27.6% (N = 51) underwent biopsy only. 95.1% (N = 176) of all patients underwent some adjuvant chemoradiation, of which 79.0% (N = 139 out of 176) underwent full adjuvant chemoradiation (Table 1).

**Table 1.** Demographic, Clinical, and Radiological Data for the Patient Cohort

Patient Data	Number	Percent
*Demographics*		
Patients	185	100.0%
Male	108	58.4%
Female	77	41.6%
Mean age at diagnosis (range)	59.5 (23.3–84.6)	
Median age at diagnosis	62.2	
*Karnofsky Performance Status*		
<70	24	13.0%
≥70	161	87.0%
*Tumor features*		
Primary glioblastoma	185	100.0%
IDH mutation (*IDH1* or *IDH2*)	18	9.7%
*MGMT* promoter methylation^a^	75	42.4%
*Extent of resection*		
Gross total resection	50	27.0%
Near-total resection	19	10.3%
Subtotal resection	65	35.1%
Biopsy	51	27.6%
*Adjuvant treatment*		
No adjuvant treatment	9	4.9%
Adjuvant treatment	176	95.1%
Full chemoradiation	139	75.1%
Partial chemoradiation	37	20.0%

^a^Censored, 177 with results.

A waterfall plot demonstrated the mutant genes found in our patient cohort sorted by mutation frequency (Figure 2). *TERT* promoter mutation (82.7% mutation frequency, N = 153), *CDKN2A* mutation (63.2%, N = 117), *CDKN2B* mutation (57.8%, N = 107), *PTEN* mutation (42.7%, N = 79), *TP53* mutation (36.8%, N = 68), and *EGFR* mutation (35.7%, N = 66) were among the most frequently mutated genes. *IDH1* mutation was found in 9.2% (N = 17) of patients.

**Figure 3.**
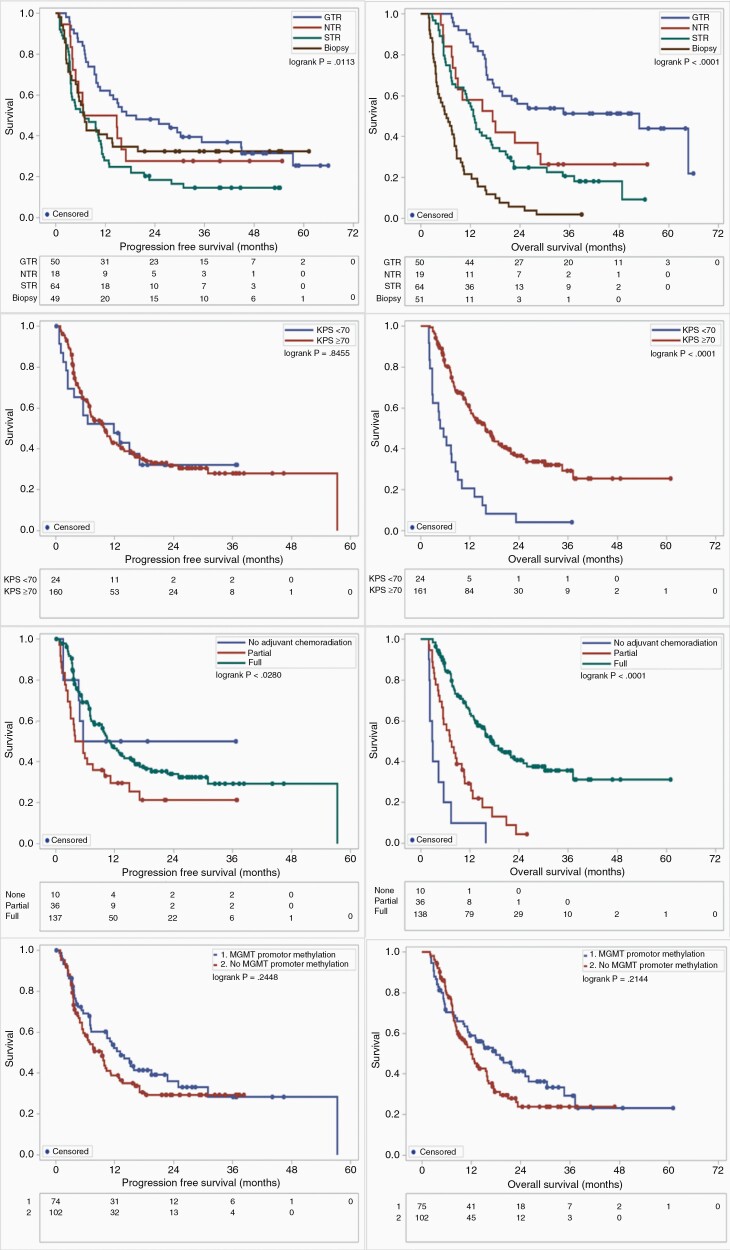
Overall survival and progression-free survival delineated by factors with known impact on prognosis. Greater extent of resection (gross total resection [GTR], near-total resection [NTR], subtotal resection [STR], and biopsy), Karnofsky Performance Status (KPS) ≥70, and extent of adjuvant chemoradiation were associated with improved overall survival. *O*^6^-methylguanine-DNA-methyltransferase (*MGMT*) promoter methylation was not associated with survival.

KM plots of factors with known impact on survival demonstrated that EOR was significantly associated with PFS (*P* = .0113) and OS (*P* < .0001). KPS ≥70 was associated with improved OS (*P* < .0001), extent of adjuvant chemoradiation was associated with improved OS (*P* < .0001), and *IDH1* or *IDH2* mutation was associated with improved OS (*P* < .0001) (Figure 3).

### Independent Prognostic Value of Gene Mutations

We performed a prognostic study using a subset of 10 genes with prognostic significance reported in the literature (Table 2). These were *TERT* promoter, *CDKN2A*, *CDKN2B*, *PTEN*, *TP53*, *EGFR*, *NF1*, *PIK3CA*, *PDGFRA*, and *IDH1*/*2*. To determine the independent prognostic value of these mutant genes compared to wildtype, we performed the analysis using covariates with known prognostic significance on survival: age, KPS, extent of adjuvant chemoradiation, *MGMT* promoter methylation status, and EOR without and with *IDH1/2* as a covariate.

**Table 2.** Independent Prognostic Value of a Subset of Commonly Mutated Genes Using Multivariate Analysis and Multiple Comparisons (N = 185)

	Progression-Free Survival				Overall Survival			
Gene mutation	*P*	FDR-adjusted * P* value	HR	95% CI	*P* value	FDR-adjusted *P* value	HR	95% CI
A								
*CDKN2A*	.0950	.3837	1.40	0.94–2.07	**.0215**	**.0430**	1.57	1.07–2.32
*CDKN2B*	.1960	.3847	1.28	0.88–1.85	**.0039**	**.0120**	1.74	1.19–2.53
*EGFR*	.1648	.3847	1.30	0.90–1.88	**.0010**	**.0100**	1.85	1.28–2.68
*NF1*	.2656	.3847	1.29	0.82–2.03	.4850	.5389	0.85	0.53–1.35
*PDGFRA*	.9788	.9788	1.01	0.54–1.88	.7427	.7427	1.10	0.61–1.99
*PIK3CA*	.5273	.6292	1.18	0.70–2.00	.3403	.4254	1.28	0.77–2.12
*PTEN*	.5663	.6292	0.90	0.62–1.29	**.0295**	**.0492**	0.66	0.46–0.96
*TERT* promoter	.1151	.3837	1.63	0.89–2.99	**.0048**	**.0120**	2.49	1.32–4.70
*TP53*	.2693	.3847	0.80	0.54–1.19	.2097	.2996	0.77	0.52–1.16
*IDH1/2*	**.0171**	.1710	0.33	0.14–0.82	**.0044**	**.0120**	0.12	0.03–0.52
Covariates: age, KPS, adjuvant chemoradiation, *MGMT* promoter methylation, EOR								
B								
*CDKN2A*	.1307	.6304	1.35	0.91–2.00	**.0320**	.0720	1.53	1.04–2.26
*CDKN2B*	.2154	.6304	1.26	0.87–1.83	**.0028**	**.0185**	1.78	1.22–2.61
*EGFR*	.3467	.6304	1.20	0.82–1.73	**.0062**	**.0186**	1.67	1.16–2.42
*NF1*	.3502	.6304	1.24	0.79–1.95	.3586	.5379	0.80	0.51–1.28
*PDGFRA*	.9915	.9915	1.00	0.54–1.87	.7030	.7030	1.12	0.62–2.02
*PIK3CA*	.5504	.8033	1.17	0.69–1.99	.4359	.5604	1.23	0.73–2.04
*PTEN*	.2441	.6304	0.80	0.56–1.16	**.0041**	**.0185**	0.58	0.40–0.84
*TERT* promoter	.6248	.8033	1.18	0.62–2.25	.1234	.2221	1.67	0.87–3.22
*TP53*	.7233	.8137	0.93	0.62–1.40	.6888	.7030	0.92	0.62–1.37
Covariates: age, KPS, adjuvant chemoradiation, *MGMT* promoter methylation, EOR, *IDH1/2* mutation								

Bolded values indicate *P* < .05.

In the FDR-adjusted OS analysis, without *IDH1*/*2* mutation as a covariate, *CDKN2A* mutation (*P* = .0430, HR 1.57), *CDKN2B* mutation (*P* = .0120, HR = 1.74), *EGFR* mutation (*P* = .0100, HR = 1.85), and *TERT* promoter mutation (*P* = .0120, HR = 2.49) were associated with decreased OS while *PTEN* mutation (*P* = .0492, HR = 0.66) and *IDH1*/*2* mutation (*P* = .0120, HR = 0.12) were associated with improved OS (Table 2, A). When adding *IDH1*/*2* mutation as a covariate to the FDR-adjusted analysis, *CDKN2B* mutation (*P* = .0185, HR = 1.78) and *EGFR* mutation (*P* = .0186, HR = 1.67) were associated with decreased OS while *PTEN* mutation (*P* = .0185, HR = 0.58) was associated with improved OS (Table 2, B). *CDKN2A* mutation was not associated with OS in the FDR-adjusted analysis with addition of *IDH1*/*2* mutation as a covariate.

In the FDR-unadjusted PFS analysis, *IDH1/2* mutation (*P* = .0171, HR = 0.33) was associated with improved PFS, but not after FDR adjustment.

Subset OS analysis of the 167 patients with *IDH1/2*-wildtype glioblastomas showed similar findings to the above analysis with *IDH1*/*2* mutation as a covariate (Supplementary Table 2). In the FDR-adjusted analysis, *CDKN2B* mutation (*P* = .0171, HR = 1.81) and *EGFR* mutation (*P* = .0231, HR = 1.65) were associated with decreased OS while *PTEN* mutation (*P* = .0171, HR = 0.58) was associated with improved OS. *CDKN2A* mutation was not significantly associated with OS in the FDR-adjusted analysis.

### Independent Prognostic Value of GTR

Next, we sought to determine the independent prognostic significance of GTR compared to all other EOR among the 10 genes previously described using age, KPS, extent of adjuvant chemoradiation, and *MGMT* promoter methylation status without and with *IDH1*/*2* as covariates. In the FDR-adjusted analysis without *IDH1*/*2* mutation as a covariate, GTR was associated with improved OS among patients with tumors harboring *CDKN2A* mutation (*P* = .0178, HR = 0.49), *CDKN2B* mutation (*P* = .0110, HR = 0.45), *EGFR* mutation (*P* = .0110, HR = 0.35), *TERT* promoter mutation (*P* = .0020, HR = 0.40), and *TP53* mutation (*P* = .0110, HR = 0.26) (Table 3, A). GTR was associated with improved OS in patients harboring *PTEN* mutation on the FDR-unadjusted analysis (*P* = .0303, HR = 0.43), but this was not significant after FDR adjustment.

**Table 3.** Independent Prognostic Value of Gross Total Resection Versus Other Extent of Resection Among Patients With Specific Gene Mutations, Using Multivariate Analysis and Multiple Comparisons

	Progression-Free Survival				Overall Survival			
Gene mutation	*P*	FDR-adjusted *P* value	HR	95% CI	*P* value	FDR-adjusted * P* value	HR	95% CI
A								
*CDKN2A*	.8471	.9387	0.95	0.57–1.58	**.0089**	**.0178**	0.49	0.28–0.83
*CDKN2B*	.9387	.9387	0.98	0.58–1.65	**.0041**	**.0110**	0.45	0.26–0.78
*EGFR*	.4060	.9387	0.76	0.40–1.45	**.0044**	**.0110**	0.35	0.17–0.72
*NF1*	.8088	.9387	1.18	0.31–4.57	.7254	.8060	0.76	0.16–3.52
*PDGFRA*	**.0441**	.2675	12.98	1.07–157.37	.2833	.3541	3.15	0.39–25.59
*PIK3CA*	.5117	.9387	0.63	0.16–2.52	.2000	.2857	0.37	0.08–1.69
*PTEN*	.7663	.9387	1.11	0.57–2.14	**.0303**	.0505	0.43	0.20–0.92
*TERT* promoter	.5856	.9387	0.88	0.56–1.38	**.0002**	**.0020**	0.40	0.25–0.65
*TP53*	.2544	.848	0.63	0.28–1.4	**.0034**	**.0110**	0.26	0.10–0.64
*IDH1/2*	.0535	.2675	28.76	0.95–869.79	.9998	.9998	0.0	0-.
Covariates: age, KPS, adjuvant chemoradiation, *MGMT* promoter methylation								
B								
*CDKN2A*	.8471	.9387	0.95	0.57–1.58	**.0224**	**.0403**	0.53	0.31–0.91
*CDKN2B*	.9387	.9387	0.98	0.58–1.65	**.0089**	**.0267**	0.48	0.28–0.83
*EGFR*	.4060	.9387	0.76	0.4–1.45	**.0044**	**.0198**	0.35	0.17–0.72
*NF1*	.8088	.9387	1.18	0.31–4.57	.7254	.7254	0.76	0.16–3.52
*PDGFRA*	**.0441**	.3969	12.98	1.07–157.37	.3039	.3907	2.99	0.37–24.03
*PIK3CA*	.5117	.9387	0.63	0.16–2.52	.4283	.4818	0.56	0.13–2.37
*PTEN*	.7663	.9387	1.11	0.57–2.14	**.0303**	**.0455**	0.43	0.20–0.92
*TERT* promoter	.5856	.9387	0.88	0.56–1.38	**.0005**	**.0045**	0.42	0.26–0.69
*TP53*	.2544	.9387	0.63	0.28–1.40	**.0135**	**.0304**	0.29	0.11–0.78
Covariates: age, KPS, adjuvant chemoradiation, *MGMT* promoter methylation, *IDH1/2* mutation								

Bolded values indicate *P* < .05.

In the FDR-adjusted analysis adding *IDH1*/*2* mutation as a covariate, GTR was associated with improved OS in patients harboring *CDKN2A* mutation (*P* = .0403, HR = 0.53), *CDKN2B* mutation (*P* = .0267, HR = 0.48), *EGFR* mutation (*P* = .0198, HR = 0.35), *PTEN* mutation (*P* = .0455, HR = 0.43), *TERT* promoter mutation (*P* = .0045, HR = 0.42), and *TP53* mutation (*P* = .0304, HR = 0.29) (Table 3, B).

In the FDR-unadjusted PFS analysis, GTR in patients harboring *PDGFRA* mutation (*P* = .0441, HR = 12.98) was associated with reduced PFS, but not after FDR-adjustment.

Patients with *IDH1/2*-wildtype glioblastomas harboring *CDKN2B*, *EGFR*, and *TERT* promoter mutations who underwent GTR were associated with improved OS when compared to other EOR in the FDR-adjusted analysis (Supplementary Table 3). *CDKN2A* mutation and *PTEN* mutation were significantly associated with improved OS in the FDR-unadjusted analysis but were no longer significant in the FDR-adjusted analysis.

### Exploratory Validation Analysis

In an attempt to validate our findings on the impact of GTR on tumor with specific mutant genes, we examined a separate institutional patient cohort (N = 108) with NGS data derived from an alternative verified platform. Mutations were categorized by varying levels of clinical significance (Supplementary Methods, Supplementary Figure 1A). Among the most frequently tested genes, the most common mutant genes were *TERT* promoter (80.6%), *PIK3CA* (40.0%), *EGFR* (38.9%), *TP53* (38.9%), *PTEN* (31.5%), and *NF1* (20.0%) (Supplementary Figure 1B). KM plots demonstrated findings similar to those found in the literature, with improved OS with greater EOR (*P* = .0025), KPS (*P* = .0001), and extent of standard adjuvant chemoradiation (*P* < .0001), and improved PFS with adjuvant chemoradiation (*P* < .0001) and *MGMT* promoter methylation (*P* = .0084) (Supplementary Figure 2). Out of the 10 genes examined in FoundationOne analysis, HR could be calculated for only 5 genes in the validation data set (*EGFR* [N = 42, 108 tested], *NF1* [N = 11, 85 tested], *PTEN* [N = 34, 108 tested], *TERT* promoter [N = 58, 72 tested], and *TP53* [N = 42, 108 tested]) because of insufficient sample size or testing of the other 5 genes (*CDKN2A* [N = 4], *CDKN2B* [N = 0], *PDGFRA* [N = 3], *PIK3CA* [N = 16, 40 tested], and *IDH1* [N = 8]).

We first ascertained if this validation cohort was sufficiently powered to detect the associations observed in our initial data set. In the bootstrapping analysis, to achieve a power of >0.80 and *P* < .05, the minimum sample sizes for each gene mutation for OS ranged from 100 to >1000 (Supplementary Table 1). In total, however, our institutional validation data set contained much fewer than 100 patients per mutation. Nevertheless, we thought it was still potentially informative to pursue an analysis of this validation cohort. We found that *PTEN* mutation was associated with improved OS if GTR was achieved without and with *IDH1/2* mutation as a covariate (*P* = .0473); however, this only trended toward significance in the FDR-adjusted analysis. *EGFR*, *NF1*, *TERT* promoter, and *TP53* mutations did not reach statistical significance in the unadjusted and adjusted analysis without *IDH1/2* mutation as a covariate (Supplementary Table 4A). We also performed a similar analysis with *IDH1/2* mutation (although *IDH2* mutation was not found in this data set) as a covariate, which showed that no single mutant gene reached statistical significance in the FDR-adjusted analysis (Supplementary Table 4B). Similarly, the results were not significant among the *IDH1/2*-wildtype group (N = 100) in the FDR-adjusted analysis (Supplementary Table 4C). Overall, in this exploratory validation analysis, although no single gene mutation was significantly associated with prognosis in the setting of GTR, there was a trend with HR < 1.0 for each of the 5 genes tested (*EGFR*, *NF1*, *PTEN*, *TERT* promoter, and *TP53*).

## Discussion

Prognostic studies are important to elucidate potential fundamental differences in glioblastoma with different molecular features. For example, *IDH1/2*-wildtype and -mutant glioblastomas have markedly different prognoses, and the WHO 2016 criteria used *IDH1/2* mutation status as a criterion when classifying glioblastomas.^26^ Several studies in the past decade have investigated the association of patient survival with mutations in genes such as *IDH1/2*, *TP53*, *PTEN*, *EGFR*, and *TERT* promoter, and more recent data show that *CDKN2A/B* deletions are associated with decreased survival when controlling for prognostic factors.^17,27,28^ For example, Shinojima et al.^14^ found that *EGFRvIII* overexpression in the setting of *EGFR* amplification was an independent marker for poor prognosis in a multivariate study. However, this study, like many others, did not take into account other significant covariates that may impact survival such as the effects of EOR and adjuvant chemoradiation, the latter of which was found to have significant implications for improved survival in more recent studies. As more information is discovered about factors with known impact on prognosis, more recent studies have begun to utilize clinical data for multivariate analyses of the prognostic significance of specific genetic mutations; however, none performed multivariate analysis while accounting for multiple gene comparisons.^17,29^ To our knowledge, this is the first study to investigate both the prognosis of tumors and the impact of EOR on glioblastoma patient outcomes using a wide spectrum of genetic events while controlling for major factors with known impact on survival. Importantly, our assessment of EOR was externally validated according to standardized criteria.^21,22^

EOR in glioblastoma has been an area of intensive study for the past 2 decades, but few studies have investigated the correlation between molecular findings of glioblastoma, EOR, and their independent impact on prognosis. For example, Felsberg et al.^30^ found that *MGMT* promoter hypermethylation and near-complete resection were independently associated with better prognosis in patients treated with resection followed by chemoradiation. A review of the current available literature suggests that GTR increases the likelihood of 1- and 2-year survival.^31^ Recent evidence also suggests that complete resection of the T1 contrast-enhancing portion in addition to a portion of the surrounding T2 hyperintensity is associated with a survival benefit over resection of the T1 contrast-enhancing portion alone in glioblastoma and that maximizing EOR may confer an additional survival advantage in patients with tumors harboring certain mutant genes including *IDH1*.^20,32^

We found that, after controlling for the effects of age, KPS, adjuvant chemoradiation, *MGMT* promoter methylation, EOR, and *IDH1/2* mutation status and accounting for multiple gene comparisons, *CDKN2B* and *EGFR* mutations were independently associated with reduced OS while *PTEN* mutation was associated with improved OS. These results validate some of the conclusions from prior studies and may provide clarification for specific mutant genes whose prognostic value is controversial.^7,15–18^ For example, the prognostic value of *PTEN* mutation has been reported to be associated with worse outcomes, in large clinical data sets,^33,34^ or to have no clear association with outcome in other studies with multivariate analyses.^35^ These studies, however, did not all control for prognostic factors such as *MGMT* methylation status, EOR, and *IDH1/2* mutation status. In addition, large studies that combine clinical and genetic data from different institutions often do not properly take into account EOR, either because the data are not available or because the definition may be nonstandardized across institutions. But our controlled results, using 6 known prognostic factors including centrally reviewed neuroradiology for EOR and FDR-adjustment, suggest that *PTEN* mutation was independently associated with *improved survival*. These findings illustrate that rigorous multivariate analysis and multiple comparisons adjustment should be performed when evaluating the effect of individual gene mutations to determine their prognostic value.

Using this approach, we also asked whether glioblastomas harboring certain genetic events were associated with survival if GTR was achieved. We found that among patients with mutations in *CDKN2A*, *CDKN2B*, *EGFR*, *PTEN*, *TERT* promoter, and *TP53*, GTR was associated with improved OS, compared to other EOR when accounting for known predictors of survival including *IDH1/2* mutation status. Moreover, in the *IDH1/2*-wildtype cohort, *CDKN2B* and *EGFR* mutations were associated with worse outcomes, but GTR still resulted in a survival benefit over other EOR.

In this study, the impact of GTR was found to be associated with 6 out of the 10 mutant genes analyzed, and this remained significant in the FDR-adjusted analysis. Since all patients in our cohort possessed at least one of these mutant genes, the result of improved survival when GTR is achieved in patients harboring these mutations is in fact a reflection of the previously known observation that GTR is beneficial to survival. Despite the common knowledge that GTR is beneficial for OS in glioblastoma patients, an intriguing hypothesis raised by our findings is that patients with tumors that do not harbor one of these 6 common mutations may not have derived benefit from GTR. However, this hypothesis remains to be verified. These findings suggest that, given the prevalence of these 6 gene mutations, following the diagnosis of glioblastoma and knowledge of *IDH1/2* mutation status, GTR (or maximum safe resection) should be attempted on all patients regardless of results from sequencing studies. The identification of druggable targets is still in its early stages, and the findings in this study suggest that patients, even those in low-resource settings, are not necessarily at a disadvantage if they do not undergo sequencing.

Although limited somewhat by sample size, this study highlights an overall approach to evaluating the contribution of genetic mutations and, in the future, other potential omic variables (such as transcriptomics, metabolomics, and proteomics) to prognosis and response to specific treatments, including surgical resection, radiation therapy, and medical therapies. This analytical framework is important for controlling for inherently heterogeneous patient populations and tumor characteristics and may have greater implications on translational studies of glioblastoma such as in clinical trial design.

Modern massively parallel DNA sequencing techniques provide a degree of granularity to glioblastoma research previously not possible by standard histopathological diagnostic methods. Unique mutations detected by NGS, such as using the FoundationOne CDx platform in this study, allow for a greater understanding of the spectrum of abnormal protein isoforms with varying degrees of clinical significance. For example, *IDH1* R132H is the most common *IDH1* mutation, but our study revealed multiple other isoforms with known and unknown clinical significance, such as R132C and R132G, which are undetectable by standard antibodies to *IDH1*.

There are several limitations to this study. This study was retrospective, performed at a single center, and is limited in power. In addition, other genes, due to their relatively low mutation frequency with insignificant *P* values in our analysis, may have an impact on clinical outcomes, but this cannot be determined given the sample size in our analysis. EOR was determined by qualitative assessment of enhancement on post-resection MRI by neuroradiology instead of by volumetric quantification of EOR. We performed a validation analysis on a separate group of 108 patients who underwent institutional NGS, and although we found that no single gene mutation was prognostic after FDR adjustment, there was a trend toward improved survival if GTR was achieved in patients harboring one of 5 common gene mutations. However, this exploratory analysis was underpowered. The findings in the current study warrant further validation using a consistent diagnostic platform and statistical analyses in a larger, multi-institutional cohort.

## Conclusion

In a single-center retrospective study of the genomic landscape of 185 glioblastoma patients, multivariate analysis controlling for 6 covariates with known impact on survival while accounting for multiple gene comparisons revealed that *CDKN2B*, *EGFR*, and *PTEN* mutations were independently associated with survival. The survival benefit of GTR was specifically seen in patients harboring mutations in *CDKN2A*, *CDKN2B*, *EGFR*, *PTEN*, *TERT* promoter, or *TP53*, which represented the entirety of the patient cohort. Therefore, the benefit of GTR seen globally in glioblastoma patients may be the result of improved survival among patients harboring any of these 6 commonly found mutant genes. These findings and our methodological approach may help to clarify results from other sequencing studies and guide future clinical and translational investigations on this topic.

## Supplementary Material

vdac002_suppl_Supplementary_Figure_S1

vdac002_suppl_Supplementary_Figure_S2

vdac002_suppl_Supplementary_Table_S1

vdac002_suppl_Supplementary_Table_S2

vdac002_suppl_Supplementary_Table_S3

vdac002_suppl_Supplementary_Table_S4

vdac002_suppl_Supplementary_Material
