# Supplementary figures and images for: Multivariate analysis of associations between clinical sequencing and outcome in glioblastoma
Source: Neurooncol Adv. 2022 Jan 10;4(1):vdac002. doi: 10.1093/noajnl/vdac002 (PMC8826782; doi:10.1093/noajnl/vdac002)

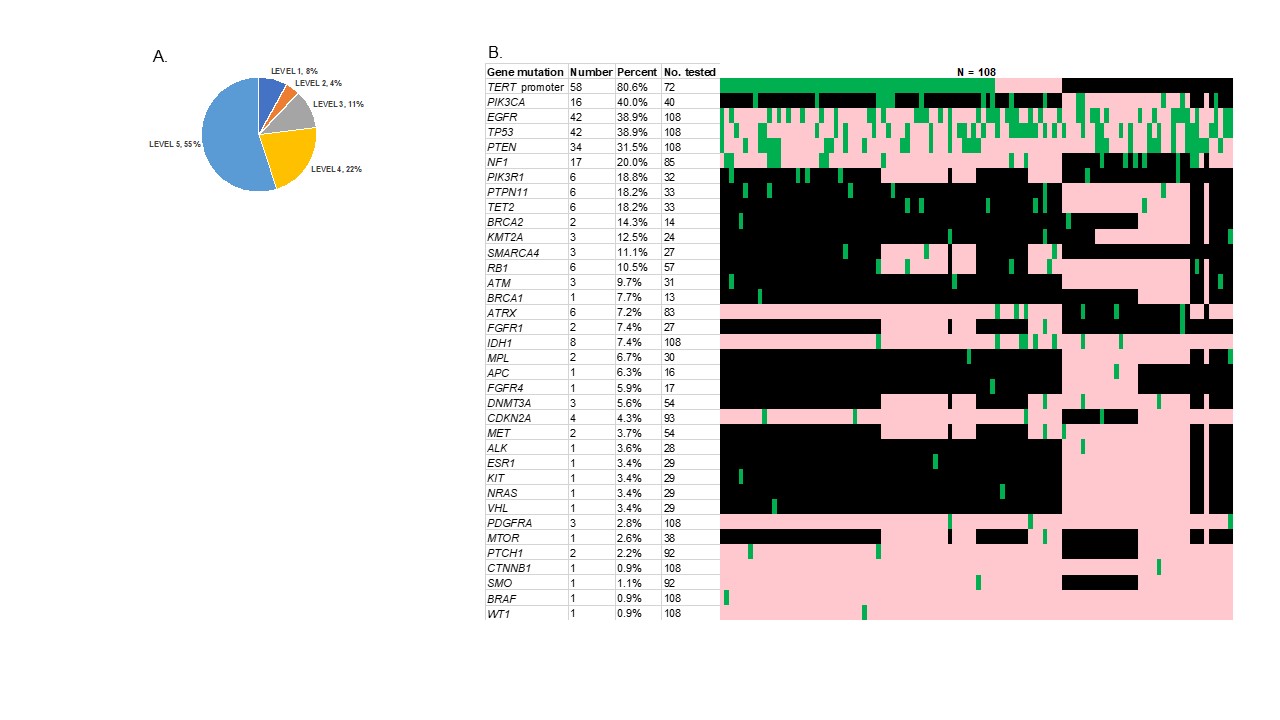

Supplement: vdac002_suppl_Supplementary_Figure_S1 [file vdac002_suppl_supplementary_figure_s1.jpeg]

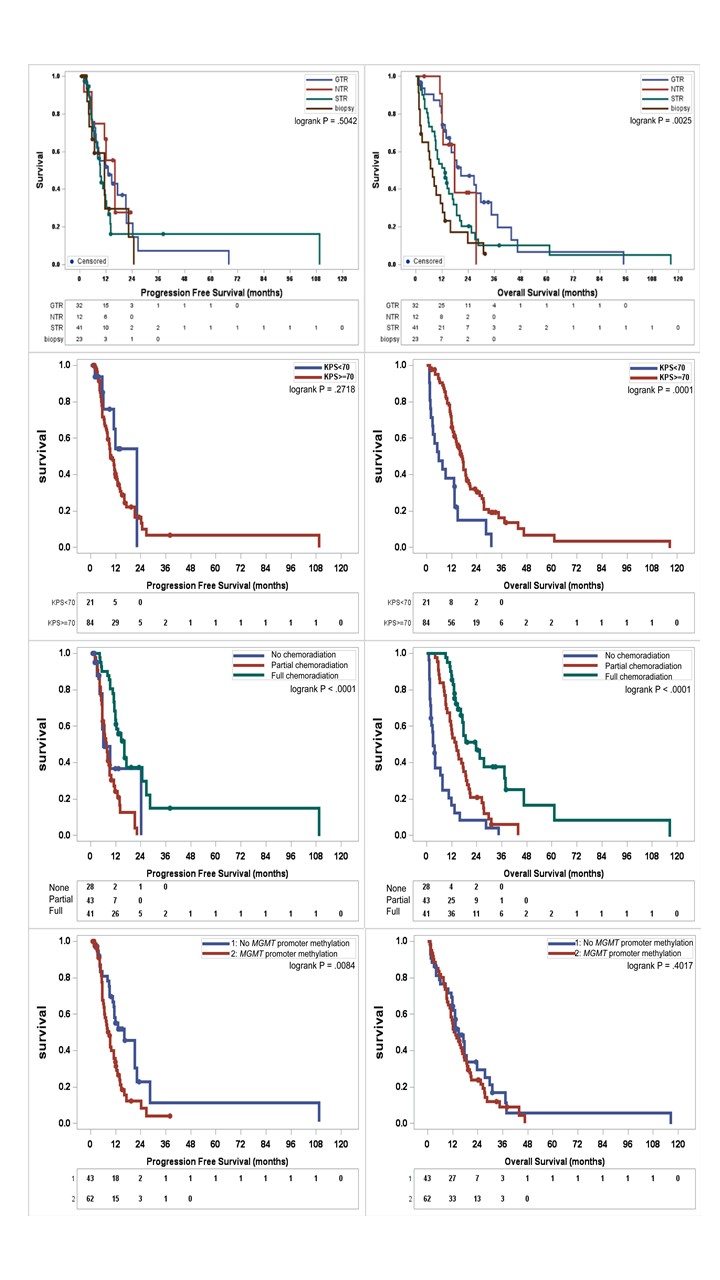

Supplement: vdac002_suppl_Supplementary_Figure_S2 [file vdac002_suppl_supplementary_figure_s2.jpeg]
