# Supplementary material for: Multivariate analysis of associations between clinical sequencing and outcome in glioblastoma
Source: Neurooncol Adv. 2022 Jan 10;4(1):vdac002. doi: 10.1093/noajnl/vdac002 (PMC8826782; doi:10.1093/noajnl/vdac002)
Supplement: vdac002_suppl_Supplementary_Table_S1 [file vdac002_suppl_supplementary_table_s1.docx]

Supplementary Table 1

Sample size calculation of the validation data set using the bootstrapping technique to calculate the number of outcome events E (progression or death) with sample size N along a predetermined sequence. Bolded numbers are P values < .05 and power > 0.80.

| **Gene** | **N** | **Survival** | **HR (mean)** | **P value (mean)** | **E** | **Power** | **E (rounded)** |
| --- | --- | --- | --- | --- | --- | --- | --- |
| EGFR | 100 | OS | 0.334005 | **0.007361** | 86.29 | **0.966667** | 86 |
| EGFR | 150 | OS | 0.330857 | **0.000499** | 129.6967 | **1** | 130 |
| EGFR | 200 | OS | 0.331384 | **6.64E-05** | 172.9967 | **1** | 173 |
| EGFR | 250 | OS | 0.330836 | **3.22E-06** | 216.0233 | **1** | 216 |
| EGFR | 300 | OS | 0.335118 | **1.24E-06** | 259.5567 | **1** | 260 |
| EGFR | 350 | OS | 0.335543 | **3.04E-07** | 302.8233 | **1** | 303 |
| EGFR | 400 | OS | 0.333216 | **1.69E-08** | 346.03 | **1** | 346 |
| EGFR | 600 | OS | 0.333296 | **2.83E-13** | 519.05 | **1** | 519 |
| EGFR | 800 | OS | 0.330567 | **4.86E-18** | 691.42 | **1** | 691 |
| EGFR | 1000 | OS | 0.329571 | **8.08E-23** | 864.0133 | **1** | 864 |
| EGFR | 100 | PFS | 0.765677 | 0.358573 | 78.78333 | 0.193333 | 79 |
| EGFR | 150 | PFS | 0.759887 | 0.287211 | 118.57 | 0.236667 | 119 |
| EGFR | 200 | PFS | 0.764641 | 0.260622 | 157.87 | 0.3 | 158 |
| EGFR | 250 | PFS | 0.762921 | 0.207218 | 197.1933 | 0.353333 | 197 |
| EGFR | 300 | PFS | 0.766926 | 0.186304 | 236.5133 | 0.416667 | 237 |
| EGFR | 350 | PFS | 0.765953 | 0.153646 | 275.9733 | 0.483333 | 276 |
| EGFR | 400 | PFS | 0.764748 | 0.127622 | 315.33 | 0.523333 | 315 |
| EGFR | 600 | PFS | 0.761615 | 0.057133 | 473.46 | 0.72 | 473 |
| EGFR | 800 | PFS | 0.762723 | **0.035599** | 630.78 | **0.833333** | 631 |
| EGFR | 1000 | PFS | 0.762629 | **0.015892** | 788.73 | **0.903333** | 789 |
| NF1 | 100 | OS | 0.818792 | 0.461406 | 83.48 | 0.036667 | 83 |
| NF1 | 150 | OS | 0.801696 | 0.403038 | 125.2 | 0.093333 | 125 |
| NF1 | 200 | OS | 0.810502 | 0.404833 | 166.9933 | 0.096667 | 167 |
| NF1 | 250 | OS | 0.806982 | 0.409066 | 208.41 | 0.116667 | 208 |
| NF1 | 300 | OS | 0.79494 | 0.391538 | 250.2133 | 0.123333 | 250 |
| NF1 | 350 | OS | 0.805148 | 0.363325 | 291.9567 | 0.146667 | 292 |
| NF1 | 400 | OS | 0.791187 | 0.342939 | 333.81 | 0.17 | 334 |
| NF1 | 600 | OS | 0.771053 | 0.263745 | 500.1167 | 0.273333 | 500 |
| NF1 | 800 | OS | 0.766839 | 0.207356 | 667.3433 | 0.39 | 667 |
| NF1 | 1000 | OS | 0.762656 | 0.154545 | 834.0567 | 0.443333 | 834 |
| NF1 | 100 | PFS | 1.259111 | 0.595607 | 77.66333 | 0 | 78 |
| NF1 | 150 | PFS | 1.245476 | 0.555984 | 116.37 | 0 | 116 |
| NF1 | 200 | PFS | 1.247679 | 0.501124 | 155.0633 | 0 | 155 |
| NF1 | 250 | PFS | 1.240669 | 0.476101 | 194.0933 | 0 | 194 |
| NF1 | 300 | PFS | 1.242042 | 0.432469 | 232.6567 | 0 | 233 |
| NF1 | 350 | PFS | 1.238662 | 0.400369 | 271.6733 | 0 | 272 |
| NF1 | 400 | PFS | 1.235068 | 0.376394 | 310.5333 | 0 | 311 |
| NF1 | 600 | PFS | 1.225246 | 0.294015 | 466.1667 | 0 | 466 |
| NF1 | 800 | PFS | 1.222182 | 0.232119 | 621.8 | 0 | 622 |
| NF1 | 1000 | PFS | 1.218092 | 0.18773 | 776.9833 | 0 | 777 |
| PTEN | 100 | OS | 0.45012 | 0.083709 | 74.46 | 0.6 | 74 |
| PTEN | 150 | OS | 0.445253 | **0.034066** | 111.6533 | **0.853333** | 112 |
| PTEN | 200 | OS | 0.439192 | **0.011126** | 149.3767 | **0.943333** | 149 |
| PTEN | 250 | OS | 0.434114 | **0.004252** | 186.5733 | **0.976667** | 187 |
| PTEN | 300 | OS | 0.433643 | **0.001985** | 223.7233 | **0.993333** | 224 |
| PTEN | 350 | OS | 0.431536 | **0.001119** | 261.1333 | **0.996667** | 261 |
| PTEN | 400 | OS | 0.429751 | **0.000165** | 298.68 | **1** | 299 |
| PTEN | 600 | OS | 0.426512 | **2.43E-06** | 447.4 | **1** | 447 |
| PTEN | 800 | OS | 0.426353 | **7.23E-08** | 597.69 | **1** | 598 |
| PTEN | 1000 | OS | 0.426213 | **9.15E-11** | 747.1333 | **1** | 747 |
| PTEN | 100 | PFS | 1.127146 | 0.560107 | 71.84333 | 0 | 72 |
| PTEN | 150 | PFS | 1.133001 | 0.524129 | 108.1967 | 0 | 108 |
| PTEN | 200 | PFS | 1.136459 | 0.511534 | 144.3033 | 0 | 144 |
| PTEN | 250 | PFS | 1.12236 | 0.518863 | 180.5433 | 0 | 181 |
| PTEN | 300 | PFS | 1.126685 | 0.497698 | 216.56 | 0 | 217 |
| PTEN | 350 | PFS | 1.124286 | 0.481157 | 252.7333 | 0 | 253 |
| PTEN | 400 | PFS | 1.122997 | 0.448981 | 288.8633 | 0 | 289 |
| PTEN | 600 | PFS | 1.123045 | 0.418897 | 432.9867 | 0 | 433 |
| PTEN | 800 | PFS | 1.120424 | 0.368154 | 577.3933 | 0 | 577 |
| PTEN | 1000 | PFS | 1.117803 | 0.336683 | 721.7767 | 0 | 722 |
| TERT | 100 | OS | 0.422689 | **0.037526** | 84.48 | **0.823333** | 84 |
| TERT | 150 | OS | 0.425445 | **0.011622** | 126.8067 | **0.953333** | 127 |
| TERT | 200 | OS | 0.424199 | **0.003699** | 169.5467 | **0.983333** | 170 |
| TERT | 250 | OS | 0.420126 | **0.003094** | 211.6667 | **0.99** | 212 |
| TERT | 300 | OS | 0.42004 | **0.000475** | 254.01 | **0.996667** | 254 |
| TERT | 350 | OS | 0.418844 | **4.35E-05** | 296.4633 | **1** | 296 |
| TERT | 400 | OS | 0.419518 | **5.39E-06** | 338.9767 | **1** | 339 |
| TERT | 600 | OS | 0.422195 | **9.34E-08** | 508.6567 | **1** | 509 |
| TERT | 800 | OS | 0.423009 | **2.34E-09** | 678.2033 | **1** | 678 |
| TERT | 1000 | OS | 0.42331 | **5.15E-13** | 847.69 | **1** | 848 |
| TERT | 100 | PFS | 0.937742 | 0.512077 | 76.76 | 0.04 | 77 |
| TERT | 150 | PFS | 0.953667 | 0.49711 | 115.6067 | 0.036667 | 116 |
| TERT | 200 | PFS | 0.953122 | 0.514581 | 154.2367 | 0.026667 | 154 |
| TERT | 250 | PFS | 0.951287 | 0.521269 | 193.02 | 0.04 | 193 |
| TERT | 300 | PFS | 0.951965 | 0.515094 | 231.8433 | 0.036667 | 232 |
| TERT | 350 | PFS | 0.949578 | 0.513226 | 270.4533 | 0.033333 | 270 |
| TERT | 400 | PFS | 0.948517 | 0.504474 | 309.17 | 0.04 | 309 |
| TERT | 600 | PFS | 0.949847 | 0.488791 | 464.76 | 0.04 | 465 |
| TERT | 800 | PFS | 0.949155 | 0.472818 | 619.5967 | 0.033333 | 620 |
| TERT | 1000 | PFS | 0.951231 | 0.483666 | 774.2267 | 0.05 | 774 |
| TP53 | 100 | OS | 0.423338 | 0.071701 | 78.48333 | 0.666667 | 78 |
| TP53 | 150 | OS | 0.403358 | **0.024724** | 117.92 | **0.883333** | 118 |
| TP53 | 200 | OS | 0.40141 | **0.009401** | 157.08 | **0.953333** | 157 |
| TP53 | 250 | OS | 0.39798 | **0.003873** | 196.1967 | **0.976667** | 196 |
| TP53 | 300 | OS | 0.396876 | **0.002188** | 235.64 | **0.99** | 236 |
| TP53 | 350 | OS | 0.395324 | **0.00064** | 274.8433 | **0.996667** | 275 |
| TP53 | 400 | OS | 0.394876 | **0.000134** | 314.29 | **1** | 314 |
| TP53 | 600 | OS | 0.390587 | **3.47E-06** | 470.4133 | **1** | 470 |
| TP53 | 800 | OS | 0.389514 | **7.28E-08** | 627.5633 | **1** | 628 |
| TP53 | 1000 | OS | 0.391095 | **7.16E-11** | 784.28 | **1** | 784 |
| TP53 | 100 | PFS | 1.014381 | 0.567442 | 70.52667 | 0.01 | 71 |
| TP53 | 150 | PFS | 1.017674 | 0.558216 | 105.63 | 0.01 | 106 |
| TP53 | 200 | PFS | 1.013069 | 0.548124 | 140.95 | 0.02 | 141 |
| TP53 | 250 | PFS | 1.007195 | 0.549923 | 176.1867 | 0.003333 | 176 |
| TP53 | 300 | PFS | 1.003274 | 0.549929 | 211.9067 | 0.006667 | 212 |
| TP53 | 350 | PFS | 0.997102 | 0.55058 | 247.0967 | 0.006667 | 247 |
| TP53 | 400 | PFS | 0.995674 | 0.562249 | 282.9367 | 0.003333 | 283 |
| TP53 | 600 | PFS | 0.990623 | 0.566148 | 423.3 | 0.006667 | 423 |
| TP53 | 800 | PFS | 0.986934 | 0.550057 | 564.6667 | 0.01 | 565 |
| TP53 | 1000 | PFS | 0.986883 | 0.537162 | 706.12 | 0.013333 | 706 |
