# Supplementary material for: Multivariate analysis of associations between clinical sequencing and outcome in glioblastoma
Source: Neurooncol Adv. 2022 Jan 10;4(1):vdac002. doi: 10.1093/noajnl/vdac002 (PMC8826782; doi:10.1093/noajnl/vdac002)
Supplement: vdac002_suppl_Supplementary_Table_S2 [file vdac002_suppl_supplementary_table_s2.docx]

Supplementary Table 2

Independent prognostic value of a subset of commonly mutated genes using multivariate analysis and multiple comparisons on *IDH1/2*-wildtype patients (N = 167)

|  | Progression-free survival | | | | Overall survival | | | |
| --- | --- | --- | --- | --- | --- | --- | --- | --- |
| Gene mutation | P value | FDR-adjusted P value | HR | 95% CI | P value | FDR-adjusted P value | HR | 95% CI |
| *CDKN2A* | .1392 | .6343 | 1.35 | 0.91--2.02 | **.0277** | .0623 | 1.56 | 1.05--2.31 |
| *CDKN2B* | .2427 | .6343 | 1.25 | 0.86--1.83 | **.0025** | **.0171** | 1.81 | 1.23--2.66 |
| *EGFR* | .3520 | .6343 | 1.19 | 0.82--1.73 | **.0077** | **.0231** | 1.65 | 1.14--2.39 |
| *NF1* | .3524 | .6343 | 1.24 | 0.79--1.95 | .3792 | .4875 | 0.81 | 0.51--1.29 |
| *PDGFRA* | .9056 | .9056 | 1.04 | 0.56--1.94 | .7197 | .7197 | 1.11 | 0.62--2.01 |
| *PIK3CA* | .6140 | .7894 | 1.15 | 0.67--1.99 | .3616 | .4875 | 1.27 | 0.76--2.12 |
| *PTEN* | .2279 | .6343 | 0.80 | 0.55--1.15 | **.0038** | **.0171** | 0.58 | 0.4--0.84 |
| *TERT* promoter | .4712 | .7068 | 1.30 | 0.64--2.63 | .1440 | .2592 | 1.66 | 0.84--3.26 |
| *TP53* | .7307 | .8220 | 0.93 | 0.61--1.41 | .6654 | .7197 | 0.92 | 0.61--1.36 |
| Covariates: age, KPS, adjuvant chemoradiation, *MGMT* promoter methylation, EOR | | | | | | | | |

Bolded values indicate P < .05
