# Supplementary material for: Multivariate analysis of associations between clinical sequencing and outcome in glioblastoma
Source: Neurooncol Adv. 2022 Jan 10;4(1):vdac002. doi: 10.1093/noajnl/vdac002 (PMC8826782; doi:10.1093/noajnl/vdac002)
Supplement: vdac002_suppl_Supplementary_Table_S3 [file vdac002_suppl_supplementary_table_s3.docx]

Supplementary Table 3

Independent prognostic value of gross total resection versus other extent of resection among *IDH1/2-*wildtype patients (N = 167) with specific gene mutations, using multivariate analysis and multiple comparisons

|  | Progression-free survival | | | | Overall survival | | | |
| --- | --- | --- | --- | --- | --- | --- | --- | --- |
| Gene mutation | P value | FDR-adjusted P value | HR | 95% CI | P value | FDR-adjusted P value | HR | 95% CI |
| *CDKN2A* | .9176 | .9506 | 1.03 | 0.61-1.72 | **.0293** | .0545 | 0.55 | 0.32-0.94 |
| *CDKN2B* | .8324 | .9506 | 1.06 | 0.62-1.8 | **.0115** | **.0345** | 0.49 | 0.29-0.85 |
| *EGFR* | .4060 | .9506 | 0.76 | 0.4-1.45 | **.0044** | **.0198** | 0.35 | 0.17-0.72 |
| *NF1* | .8088 | .9506 | 1.18 | 0.31-4.57 | .7254 | .7254 | 0.76 | 0.16-3.52 |
| *PDGFRA* | **.0488** | .4392 | 12.60 | 1.01-156.75 | .3039 | .3907 | 2.99 | 0.37-24.03 |
| *PIK3CA* | .7740 | .9506 | 0.81 | 0.19-3.46 | .4283 | .4818 | 0.56 | 0.13-2.37 |
| *PTEN* | .7663 | .9506 | 1.11 | 0.57-2.14 | **.0303** | .0545 | 0.43 | 0.20-0.92 |
| *TERT* promoter | .8073 | .9506 | 0.95 | 0.61-1.48 | **.0005** | **.0045** | 0.42 | 0.26-0.69 |
| *TP53* | .9506 | .9506 | 0.97 | 0.41-2.33 | .0816 | .1224 | 0.40 | 0.14-1.12 |
| Covariates: age, KPS, adjuvant chemoradiation, *MGMT* promoter methylation | | | | | | | | |

Bolded values indicate P < .05
