# Supplementary material for: Multivariate analysis of associations between clinical sequencing and outcome in glioblastoma
Source: Neurooncol Adv. 2022 Jan 10;4(1):vdac002. doi: 10.1093/noajnl/vdac002 (PMC8826782; doi:10.1093/noajnl/vdac002)
Supplement: vdac002_suppl_Supplementary_Table_S4 [file vdac002_suppl_supplementary_table_s4.docx]

| A. | Progression-free survival | | | | Overall survival | | | |
| --- | --- | --- | --- | --- | --- | --- | --- | --- |
| Gene mutation | P value | FDR-adjusted P value | HR | 95% CI | P value | FDR-adjusted P value | HR | 95% CI |
| *EGFR* | 0.7071 | 0.8839 | 0.76 | 0.19-3.09 | 0.5896 | 0.5896 | 0.65 | 0.13-3.14 |
| *NF1* | 0.9975 | 0.9975 | 0.00 | 0.00-. | 0.1401 | 0.3503 | 0.01 | 0.00-5.10 |
| *PTEN* | 0.5084 | 0.8473 | 0.66 | 0.20-2.23 | **0.0473** | 0.2365 | 0.27 | 0.08-0.98 |
| *TERT* promoter | 0.1216 | 0.3040 | 0.43 | 0.15-1.25 | 0.2376 | 0.3960 | 0.56 | 0.22-1.46 |
| *TP53* | 0.1015 | 0.3040 | 0.36 | 0.11-1.22 | 0.3416 | 0.4270 | 0.61 | 0.22-1.70 |
| Covariates: age, KPS, adjuvant chemoradiation, *MGMT* promoter methylation | | | | | | | | |
|  |  |  |  |  |  |  |  |  |
| B. | Progression-free survival | | | | Overall survival | | | |
| Gene mutation | P value | FDR-adjusted P value | HR | 95% CI | P value | FDR-adjusted P value | HR | 95% CI |
| *EGFR* | 0.7403 | 0.9254 | 0.79 | 0.2-3.17 | 0.4186 | 0.4186 | 0.51 | 0.10-2.59 |
| *NF1* | 0.9984 | 0.9984 | 0.00 | 0.00-. | 0.1326 | 0.2238 | 0.01 | 0.00-4.22 |
| *PTEN* | 0.5084 | 0.8473 | 0.66 | 0.20-2.23 | **0.0473** | 0.2238 | 0.27 | 0.08-0.98 |
| *TERT* promoter | 0.2045 | 0.5113 | 0.49 | 0.16-1.47 | 0.2602 | 0.3253 | 0.58 | 0.22-1.50 |
| *TP53* | 0.1066 | 0.5113 | 0.35 | 0.10-1.25 | 0.1343 | 0.2238 | 0.44 | 0.15-1.29 |
| Covariates: age, KPS, adjuvant chemoradiation, *MGMT* promoter methylation, *IDH1/2* mutation | | | | | | | | |
|  |  |  |  |  |  |  |  |  |
| C. | Progression-free survival | | | | Overall survival | | | |
| Gene mutation | P value | FDR-adjusted P value | HR | 95% CI | P value | FDR-adjusted P value | HR | 95% CI |
| *EGFR* | 0.6298 | 0.7873 | 0.71 | 0.17-2.89 | 0.4988 | 0.4988 | 0.57 | 0.12-2.87 |
| *NF1* | 0.9973 | 0.9973 | 0.00 | 0.00-. | 0.1326 | 0.2210 | 0.01 | 0.00-4.22 |
| *PTEN* | 0.5084 | 0.7873 | 0.66 | 0.20-2.23 | **0.0473** | 0.1550 | 0.27 | 0.08-0.98 |
| *TERT* promoter | 0.2046 | 0.5115 | 0.49 | 0.16-1.47 | 0.2603 | 0.3254 | 0.58 | 0.22-1.50 |
| *TP53* | 0.0857 | 0.4285 | 0.27 | 0.06-1.20 | 0.0620 | 0.1550 | 0.32 | 0.10-1.06 |
| Covariates: age, KPS, adjuvant chemoradiation, *MGMT* promoter methylation | | | | | | | | |

Supplementary Table 4

Independent prognostic value of gross total resection versus other extent of resection among patients with specific gene mutations, using multivariate analysis and multiple comparisons for the validation data set (N = 108).

A. Analysis with covariates: age, KPS, adjuvant chemoradiation, *MGMT* promoter methylation

B. Analysis with addition of *IDH1/2* mutation as a covariate

C. Analysis of only *IDH1/2*-wildtype patients (N = 100)

Bolded values indicate P < .05
