# Supplementary material for: Multivariate analysis of associations between clinical sequencing and outcome in glioblastoma
Source: Neurooncol Adv. 2022 Jan 10;4(1):vdac002. doi: 10.1093/noajnl/vdac002 (PMC8826782; doi:10.1093/noajnl/vdac002)
Supplement: vdac002_suppl_Supplementary_Material [file vdac002_suppl_supplementary_material.docx]

Supplementary Methods for Validation Cohort

We collected clinical and radiologic data from glioblastoma patients who underwent surgery from 2011-2015 for a total of 108 glioblastoma patients. Data collected from these 108 patients included age, Karnofsky Performance Status (KPS), extent of resection (EOR), *MGMT* promoter methylation status, adjuvant chemoradiation status, progression-free survival (PFS), and overall survival (OS). All patients underwent post-operative MRI within 72 hours of surgery and had external neuroradiology validation of EOR. Tumor samples from these patients underwent next generation sequencing (NGS) using a previously validated assay.^1-3^

*Power analysis*

We first sought to determine whether we had adequate sample size in our validation data set per gene mutation in the multivariate setting (power analysis). We used the bootstrapping technique on the FoundationOne data set using the following steps:

(1) Set a bootstrap data set of sample size of N gene mutations. The number of outcome events E (progression or death) was calculated, with N fixed across bootstrap replicates while E varying across bootstrap replicates.

(2) Perform the same multivariate Cox model on the bootstrap data set to estimate hazard ratio (HR) and the Wald test P value.

(3) Assign a flag of 1 if HR < 1 (GTR vs. other EOR with a Wald test P < .05) or assign a flag of 0 if the condition is not met.

(1) to (3) were repeated to obtain 300 bootstrap replicates per gene and per sample size N to calculate the power at the sample size N. The average number of events E across the bootstrap replicates was also calculated. N was evaluated along a predetermined sequence, N = (100, 150, 200, 250, 300, 350, 400, 600, 800, 1000), and the power was calculated for PFS and OS for each gene (Supplementary Table 1).

This power analysis was run using the 10 genes examined in the main text (*CDKN2A*, *CDKN2B*, *EGFR*, *NF1*, *PDGFRA*, *PIK3CA*, *PTEN*, *TERT* promoter, and *TP53*). However, *CDKN2B* could not be found in the validation data set, and HRs could not be calculated for *CDKN2A*, *PDGFRA*, *PIK3CA*, and *IDH1* due to small sample sizes so these five genes were omitted from further analysis. Therefore, *EGFR*, *NF1*, *PTEN*, *TERT* promoter, and *TP53* were used in this validation analysis.

*Mutation calling*

As per the standard protocol at our institution during this period of time,^1-3^ genomic DNA mutation data were categorized into five pre-determined levels by the neuropathologist with varying levels of clinical significance. Level 1 variants were predictive or prognostic in glioblastoma. Level 2 variants were predictive or prognostic in other tumor types. Level 3 variants were reported in cancer or other disease. Level 4 variants were variants of uncertain clinical significance. Level 5 variants were known polymorphisms (wildtype). We acknowledge that certain gene variants may not fit in “level 1” or “level 2” exclusively. Therefore, we have grouped levels 1-3 as “mutant” as the distinction between these levels may be more fluid than the distinction between levels 1-3 vs. level 4 vs. level 5. Thus, for this analysis, levels 1-3 were considered to be mutant (23%) while level 5 (55%) was considered to be wild-type (Supplementary Figure 1A). Variants of uncertain clinical significance, or level 4, were excluded from the analysis due to their currently unknown significance. A waterfall plot is shown in Supplementary Figure 1B.

*Statistics*

Kaplan-Meier (KM) analysis was conducted to generate survival curves for PFS and OS of parameters with previously known impact on survival. The log-rank test was used to examine the statistical significance of the differences observed between the groups. A prognostic study was performed using a multivariate Cox proportional hazards model to compute HRs and 95% confidence intervals of the most frequently mutated tumor genes while controlling for covariates with known impact on survival. Finally, a multivariate analysis was performed to investigate the impact on survival of gross total resection vs. all other EOR in patients with tumors that harbored the most frequently mutated genes. The Benjamini-Hochberg procedure controlling false discovery rate (FDR) at a 5% level was used to adjust the survival analysis. The two-tailed t test with P < .05 was considered statistically significant. All analyses were performed within Statistical Analysis System (SAS) (version 9.4; SAS, Cary, NC, USA).

References:

**1.** Cottrell CE, Al-Kateb H, Bredemeyer AJ, et al. Validation of a next-generation sequencing assay for clinical molecular oncology. *The Journal of molecular diagnostics.* 2014; 16(1):89-105.

**2.** McNulty SN, Cottrell CE, Vigh-Conrad KA, et al. Beyond sequence variation: assessment of copy number variation in adult glioblastoma through targeted tumor somatic profiling. *Human pathology.* 2019; 86:170-181.

**3.** Carter JH, McNulty SN, Cimino PJ, et al. Targeted next-generation sequencing in molecular subtyping of lower-grade diffuse gliomas: application of the World Health Organization's 2016 revised criteria for central nervous system tumors. *The Journal of Molecular Diagnostics.* 2017; 19(2):328-337.
